# The Atypical Presentation of Ifosfamide-Induced Renal Tubular Acidosis

**DOI:** 10.7759/cureus.63862

**Published:** 2024-07-04

**Authors:** Vlad Vayzband, Michael Mira, Karlene Williams

**Affiliations:** 1 Internal Medicine, Overlook Medical Center, Summit, USA

**Keywords:** chemotherapy, drug-induced hypokalemia, renal tubular acidosis, drug toxicity, ifosfamide

## Abstract

Antineoplastic agents are often associated with a wide range of side effects, caused by either direct toxicity or indirect through their metabolism. Ifosfamide is a cytotoxic, antineoplastic medication that is known to cause a direct tubular injury with an associated normal anion gap metabolic acidosis due to type 1 or type 2 renal tubular acidosis (RTA). The manifestations and approach to its diagnosis have been well established. However, we present a case in which a patient presented with acute symptomatic hypokalemia in the setting of ongoing ifosfamide use for metastatic osteosarcoma but without the typical laboratory findings. The clinical- and laboratory-driven diagnosis of suspected type 3 renal tubular acidosis involving proximal and distal segments is suggested by this case report.

## Introduction

Ifosfamide is an alkylating agent that has been approved for the treatment of different cancers in adults and children. It is a synthetic analog of cyclophosphamide and is primarily excreted in the urine. Ifosfamide is known to cause kidney injury, leading to many unfavorable manifestations due to tubular dysfunction. The most well-known are Fanconi syndrome, acute tubular acidosis (ATN), hypophosphatemia, and metabolic acidosis with normal anion gap due to type 1 or type 2 renal tubular acidosis (RTA) resulting in hypokalemia. Potassium is an incredibly important intracellular cation that is maintained within normal physiologic parameters by multiple internal feedback mechanisms. The normal serum potassium range is 3.5-4.5 mmol/L. Notably, this is the serum potassium and not the intracellular potassium concentration. This elicits clinical importance in settings of hypokalemia. Since serum potassium functions as a surrogate, which often underestimates the intracellular concentration, for every 1 mEq/L decrease from an individual’s established homeostatic baseline, a required 200-400 mEq of potassium is needed [1-3]. Manifestations of hypokalemia occur once serum values are less than 3.0 mmol/L, including cardiac arrhythmias and muscle weakness beginning in the lower extremities and progressing superiorly, potentially involving the respiratory muscles [3]. Given the high mortality potential, physiologic mechanisms to increase potassium occur systemically through increased sympathetic output but arguably most importantly through the kidney’s ability to regulate the potassium and acid-base status. Thus, in instances when hypokalemia is suspected, it is vital to ascertain the cause, replete the deficit appropriately, and ensure that the renal function remains appropriate. Herein, we present a case in which the body’s innate defense to hypokalemia was impaired, and this led to an atypical presentation of an otherwise typical pathology.

## Case presentation

**Figure 1:**
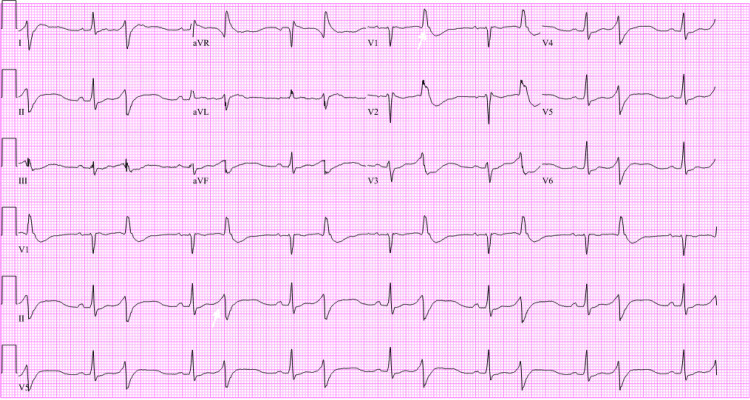
EKG showing sinus rhythm with frequent premature ventricular complexes in a pattern of bigeminy as exemplified by the white arrow in the lead 2 rhythm strip. There is an incomplete right bundle branch block as denoted by the white arrow in lead V1. The QT/QTc interval is 526/618 ms. QTc: corrected QT

A 24-year-old patient with a history of recurrent metastatic osteosarcoma status post-surgical and radiologic interventions and with ongoing chemotherapy with etoposide and ifosfamide presented with symptomatic hypokalemia while undergoing outpatient chemotherapy. The patient endorsed diffuse muscle spasms and weakness, which culminated in a witnessed syncopal episode, likely due to significant bradycardia. At the onset of symptoms, potassium was found to be 2.3, below their baseline range of 2.9-3.2, which was repleted with a total of 80 mEq of potassium. Despite resuscitative efforts in the outpatient setting, repeat potassium was 1.8, prompting the hospital visit. On admission, laboratory results revealed a potassium of 1.3 and significant bradycardia with a heart rate of 43 as determined by manual palpation and a prolonged corrected QT (QTc) with bigeminy (Table 1 and Figure 1). Phosphorus was notably 0.4 and magnesium 1.8 (Table 1). Venous blood gas revealed a normal pH and a normal serum bicarbonate of 25 (Table 2). Urinalysis was significant for a urine pH of 6.0, glucosuria of >1000, and a urine anion gap of 9 (Table 3). The patient was then transferred to the ICU for closer monitoring. The patient received a total of 345 mEq of potassium over the course of two days, which increased serum potassium to ranges between 3.1 and 4.6. Magnesium and other electrolytes were also repleted to normal serum values.

**Table 1:** Basic metabolic panel (BMP). Serum values are seen from the day of admission to the day of discharge, denoted as 18 hours following presentation. It is notable for normal serum bicarbonate levels and chloride. Upon discharge and follow-up, serum potassium returned to baseline values of 2.9-3.2.

BMP	Reference	Presentation	6 Hours After Presentation	12 Hours After Presentation	18 Hours After Presentation	Outpatient One Week Following Discharge
Sodium	135-145 mEq/L	142	142	145	146	141
Potassium	3.5-4.5 mEq/L	1.3	1.9	3.1	4.6	3.0
Chloride	98-108 mEq/L	110	112	114	121	101
Carbon Dioxide	22-28 mEq/L	24	25	25	22	25
Creatinine	0.6-1.2 mg/dL	1.25	1.06	1.12	0.95	1.47
Glucose	70-110 mg/dL	171	156	126	92	96
Phosphorus	3.0-4.5 mg/dL	0.4	1.2	3.6	3.2	1.8
Magnesium	1.5-2.0 mEq/L	1.8	2.9	2.5	2.3	2.2

**Table 2:** Venous blood gas. Venous blood gas drawn at presentation represents a normal pH. pCO_2_, partial pressure of carbon dioxide; HCO_3_, bicarbonate; pO_2_, partial pressure of oxygen

Venous Blood Gas	Reference	Presentation
pH	7.31-7.41	7.38
pCO_2_	41-51 mmHg	36
HCO_3_	23-29 mmol/L	21.2
pO_2_	30-40 mmHg	50

**Table 3:** Urine analysis. It is notable for glucosuria, alkalotic range pH, and a clinically indeterminate urine anion gap. The urine anion gap is not significantly elevated enough to be considered positive.

Urinalysis	Reference	Presentation
Glucose	0-0.8 mmol/L	>1000
pH	4.5-8.0	6.0
Specific Gravity	1.005-1.030	1.008
Sodium	>20 mEq/L	72
Potassium	25-100 mEq/Day	14
Chloride	140-250 mEq/Day	77
Urine Anion Gap	Variable to Above Levels	+9

## Discussion

The evaluation of an arrhythmia with associated muscle spasms often involves serum electrolyte disturbances. As a presenting symptom, which will often be accompanied by hypokalemia, the understanding of potassium distribution is important. Generally, the underlying mechanism can be divided into categories, which include transcellular shifts, which are primarily regulated by the sodium-potassium ATPase and hydrogen-potassium ATPase (internal balance), renal potassium loss often in the setting of dysregulations in tubular function, extrarenal losses as is often seen in settings of vomiting and diarrhea, and lastly through reduced dietary intake (external balance). As a further simplification, in most instances, the inciting cause is driven by either gastrointestinal or renal loss. In both instances, potassium wasting will be accompanied by an acid-base disturbance.

When evaluating our patient, it was clear that they had severe hypokalemia, but the etiology was not as evident. Based on the basic metabolic panel (BMP), no obvious acidosis was present. On presentation, there was a normal anion gap with no elevation of chloride. Typically, when acidosis is suspected, a blood gas would confirm the suspicion; however, the venous blood gas was also within normal limits. Although no potassium creatinine ratio was calculated, the patient did not endorse any gastrointestinal disturbances to assume extrarenal losses. Blood pressures at the time of presentation and throughout the hospital course remained within the systolic range of 90-110, further excluding other potential causes such as the adrenals, renal vasculature, or primary hyperaldosteronism. In most instances, the acid-base status would provide the highest-yielding diagnostic clue to the underlying etiology. However, it was grossly unremarkable. At the very least, what could be said was that the potential causes of either metabolic alkalosis or acidosis would be entirely renally driven; that much could be said for certain. Based on the patient’s history, there was no reported genetic or ongoing hypokalemia before the initiation of ifosfamide to suspect an inherited tubulopathy, broadly then excluding conditions such as Bartter and Gitelman syndromes. Therefore, the most likely cause of this acute hypokalemia in the setting of ongoing ifosfamide use would be renal tubular acidosis. However, we were missing the acidosis.

Since the proximal renal segments are responsible for bicarbonate creation and the distal portions for bicarbonate reabsorption, any disturbance in these areas should result in a decreased blood pH. Additionally, the proximal segments are responsible for the highest proportion of the electrolytes and glucose reabsorbed in the nephron. Hence, a decline in serum phosphorus, bicarbonate, potassium, and elevated glucose within the urine is expected when the proximal segment is dysfunctional. Likewise, when the role of the distal segment is impaired, it is expected to see a reduced serum bicarbonate and potassium but no glucosuria. The urine anion gap and pH then inform us which segment specifically is affected. Since the urine anion gap is a surrogate for ammonium concentration, when the proximal portion is affected, the negatively charged ammonia will not be exchanged for positively charged ammonium, leading to a negative urine anion gap and a pH of less than 5.3. Therefore, if the urine anion gap is elevated beyond the value of 20 in a urine pH greater than 5.3, it indicates appropriate ammonium generation but with an inability to acidify the urine, which is commonly seen in distal tubulopathies. Our patient, however, had a urine anion gap of <20 and a pH of >5.3, suggesting reduced ammonium generation and an inability to acidify the urine. When these findings are coupled with other supportive features for a primary proximal tubulopathy, which included hypophosphatemia, hypokalemia, and glucosuria, the diagnosis is almost certain, but we are still missing the acidosis.

Ifosfamide has been well established to induce proximal tubulopathies, but not much literature has been found regarding its involvement in distal tubulopathies and, as this case report suggests, concomitant tubulopathies [1-5]. Based on the evidence collected, we suggest that this patient had a mixed proximal and distal tubulopathy induced by ifosfamide. The current understanding regarding ifosfamide toxicity involves direct mitochondrial damage [1,2,6]. It is believed to be taken up by the influx carrier human organic cation transporter 2, which is in segments of high mitochondrial density [1,2,4,7]. Research that looked at renal damage in individuals taking ifosfamide showed that proximal segments are most affected with electron microscopic imaging showing signs specific to mitochondrial damage [1,2,4]. Both the proximal and distal segments have a high density of both mitochondria and the transporter [2,4].^ ^Although we cannot say this is the exact pathological process that was seen in this patient, we do suspect that there is something intrinsic and highly specific to these two segments for the pathological process to only occur there.

## Conclusions

Hypokalemia is an important and potentially devastating side effect of ifosfamide use. It is an established cause of proximal tubulopathies and, from this case presentation, may also be involved in the disruption of more distal segments, suggesting a diagnosis of type 3 renal tubular acidosis. More research is needed to establish this involvement. However, the manifestations and treatment of hypokalemia must be dealt with promptly, remembering that potassium repletion is aimed at intracellular rather than extracellular serum potassium.
